# Area from image analyses accurately estimates dry‐weight biomass of juvenile tissue from the moss *Ceratodon purpureus*

**DOI:** 10.1002/aps3.11418

**Published:** 2021-04-30

**Authors:** Wesley P. Burtscher, Marna A. List, Adam C. Payton, Stuart F. McDaniel, Sarah B. Carey

**Affiliations:** ^1^ Department of Biology University of Florida Gainesville Florida 32611 USA

**Keywords:** *Ceratodon purpureus*, development, ImageJ, moss, phenotype, *Physcomitrella (Physcomitrium) patens*, protonema, tissue culture

## Abstract

**PREMISE:**

Mosses have long served as models for studying many areas of plant biology. Investigators have used two‐dimensional measurements of juvenile growth from photographs as a surrogate for dry‐weight biomass. The relationship between area and biomass, however, has not been critically evaluated.

**METHODS:**

Here we grew axenic tissue cultures of 10 *Ceratodon purpureus* isolates to study the relationship between these parameters. We measured area and biomass on replicate cultures with two distinct starting inoculum sizes each week for three weeks. We then examined the correlation between area and biomass as well as the influence of variation in inoculum size on both parameters.

**RESULTS:**

We found a strong correlation between area and biomass after two weeks of growth. Furthermore, we found inoculum size affected biomass during the first week of growth but not in subsequent weeks and inoculum size had no detectable effect on area.

**DISCUSSION:**

These analyses provide experimental confirmation that area is a suitable proxy for biomass and provide clear guidelines for when inoculum size variation may affect downstream growth estimates.

Mosses have long served as models for studying many problems in plant biology (Cove, 2005; Prigge and Bezanilla, 2010). One of the reasons for their utility is that the juvenile moss tissue, composed of protonema, is easy to clonally propagate in sterile conditions. Protonema grow in filaments comprised of two cell types (chloronema and caulonema) that divide by serial extension and frequently branch, forming a dense network of tissue. The maturation of protonema triggers the production of buds, which begin to divide along multiple planes, producing mature, leafy gametophores. It is relatively simple to generate axenic protonemal tissue from germinating spores, wounded mature gametophore tissue, or by subsampling or blending growing protonema (Cove et al., 2009a). The cell walls of protonema can also be digested to generate abundant protoplasts (Cove et al., 2009b). In addition, protonema are responsive to hormone treatments, as well as other chemical or environmental perturbations, and are amenable to ultraviolet and chemical mutagenesis (Cove et al., 2009c). The fact that identical haploid clonal replicates can be exposed to the same experimental manipulations in a relatively small amount of space, like a growth chamber, makes moss protonema particularly useful.

As a consequence of these traits, many investigators have used protonemal growth rate as a focal phenotype or a surrogate for fitness (McDaniel et al., 2008; Nomura and Hasezawa, 2011; Proust et al., 2011; Tani et al., 2011; Cho et al., 2012; Rawat et al., 2017). The standard practice is to photograph protonemal tissue growing on agar‐containing Petri dishes and measure the area occupied by a particular clone. This approach, however, uses a two‐dimensional representation of a three‐dimensional phenotype, because protonema grow orthogonal to the surface of the agar (i.e., up) as well as laterally. Thus, two genotypes with similar growth rates but different growth patterns (primarily horizontal growth versus primarily vertical growth) could be erroneously scored as having different biomasses using the typical image analysis procedure. Such variation in growth form is not uncommon among natural isolates or mutagenized plants (McDaniel et al., 2008; Perroud and Quatrano, 2008). Thus, dry‐weight biomass may provide a better estimate of overall growth than does two‐dimensional area. Measuring protonema using dry‐weight biomass, however, cannot be easily automated, making it a time‐consuming procedure, and it can only be measured in effectively dead cells, which negates some of the benefits of working with moss protonema.

In this paper, we describe a critical evaluation of the relationship between the two‐dimensional area of protonema, estimated from photographs, and the more biologically relevant trait of biomass. We grew 10 diverse isolates of the model moss *Ceratodon purpureus* (Hedw.) Brid. and measured both two‐dimensional area and biomass at three time points. We used isolates representing the global diversity of *C*. *purpureus* because the species is highly polymorphic in growth rate and other life history traits (Shaw and Beer, 1999; McDaniel, 2005). We found a strong correlation between area and biomass after two weeks of growth. We also varied the starting inoculum size to evaluate how sensitive the two measures of protonemal growth were to such variation. This measure may be important in labs where multiple individuals initiate experimental cultures or when the starting size of the inoculum varies because of other experimental variables. Although we could detect an effect of inoculum size on biomass after one week, we found no effect after two weeks. Moreover, we detected no effect of inoculum size on area after only one week. Collectively, these data indicate that area is a suitable proxy for dry‐weight biomass, and that for *C. purpureus* growth experiments the effect of inoculum size variation disappears after two weeks.

## MATERIALS AND METHODS

### Tissue generation

The *C*. *purpureus* isolates used in this study were previously grown from single‐spore isolates and maintained as lab cultures. We used five male/female sibling pairs (i.e., a male and female from the same sporophyte) that were originally isolated from different habitats in Ecuador, Chile, New York, Connecticut, and Alaska (tropics, high arctic, and temperate zones). To generate tissue for testing area against biomass and the effect of starting inoculum size, we grew axenic tissue cultures following Cove et al. (2009a). We divided the plates into two inoculum treatments: small (1–2 mm in diameter) and large (3–4 mm, or approximately twice the small inoculum size). For both inoculum sizes, the moss tissue was rolled into visually uniform balls using tweezers under a dissecting microscope; as many replicates as possible were prepared at once to maintain a consistent size. Each time point had 15 Petri dishes containing four isolates (although a few only had two isolates). Each isolate was replicated six times per time point and each replicate was placed on a randomly selected plate. Three clonal replicates of each isolate were used per time point for the large inoculum size analyses and each isolate was grown on a randomly selected plate. The same tissue for the inoculum‐size analyses was used for comparisons of area and biomass. The tissue was grown on cellulose cellophane disks overlaying 0.7% agar (A9799, Plant Cell Culture Agar; Sigma‐Aldrich, St. Louis, Missouri, USA) in 100‐mm‐diameter Petri dishes containing BCD medium and 5 mM (di)ammonium tartrate (Cove et al., 2009a). Cultures were grown at a constant temperature of 25°C in 18‐h full light days in Percival Scientific growth chambers (Percival Scientific Inc., Perry, Iowa, USA) with a light intensity of 60–80 μmol m^−2^ s^−1^. We haphazardly rotated plate positions inside the growth chamber every 24 h.

### Data collection

Measurements of area and biomass of the small and large inoculum sizes were taken at one, two, and three weeks of growth. To collect area measurements, each plate was photographed using a Canon EOS 50D camera with a 50‐mm lens (Canon Inc., Ota City, Tokyo, Japan) and assessed using ImageJ (Schneider et al., 2012). ImageJ measures area by converting pixels to square millimeters using the area of the plate itself as a calibration. To collect biomass, we harvested the designated replicates at each time point and placed each separately into a 1.5‐mL tube, which we had measured beforehand so that the study controlled for variation in tube weight. The tissue was then dried in a precision gravity convection incubator for four days at 37°C. We weighed the dried tissue to the 0.1 mg accuracy on a Mettler Toledo balance (model no. XS64; Mettler Toledo, Columbus, Ohio, USA).

The final data set included 264 replicates—between 25 and 27 for each of 10 isolates. We removed two clonal replicates that died; several others showed signs of bacterial contamination, but we have included these in our analyses.

### Statistical analyses

All statistical analyses and plots were done in Base R (version 3.4.2; R Core Team, 2017). To test whether area, a two‐dimensional representation of moss growth, is an acceptable proxy for biomass, we first averaged biomass and area for all clonal replicates within each inoculum size for each single‐spore isolate and ran separate correlations for each week using cor(). To test whether starting inoculum size of protonemal tissue affects the results of area and biomass measurements, we ran ANOVAs on each week separately using aov().

## RESULTS

**FIGURE 1:**
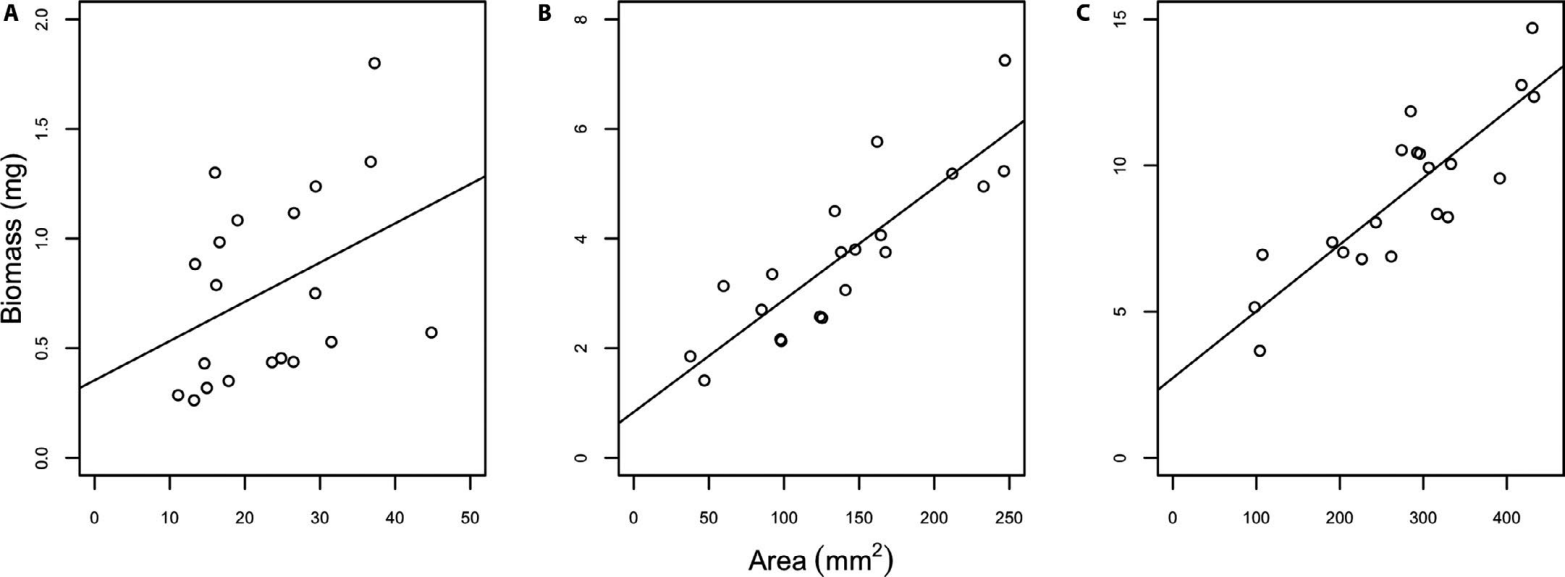
Scatterplots of mean biomass (in milligrams) and mean area (in square millimeters) during three weeks of growth in large and small inoculum sizes of 10 isolates of *Ceratodon purpureus*. Area and biomass were compared at one (A), two (B), and three (C) weeks of growth. Week 1 shows a weak positive correlation (*r* = 0.393), whereas weeks 2 (*r* = 0.858) and 3 (*r* = 0.837) show a strong positive correlation. Lines are linear least‐squares regression fits to data points.

To test the strength of the relationship between change in area and change in biomass, we ran a correlation of area by biomass for each week’s data. We found a weak correlation between area and biomass for week 1 (*r* = 0.393; Fig. 1A) but strong correlations between the two variables in weeks 2 and 3 (0.858 and 0.837, respectively; Fig. 1B, C; data in Appendix S1).

**FIGURE 2:**
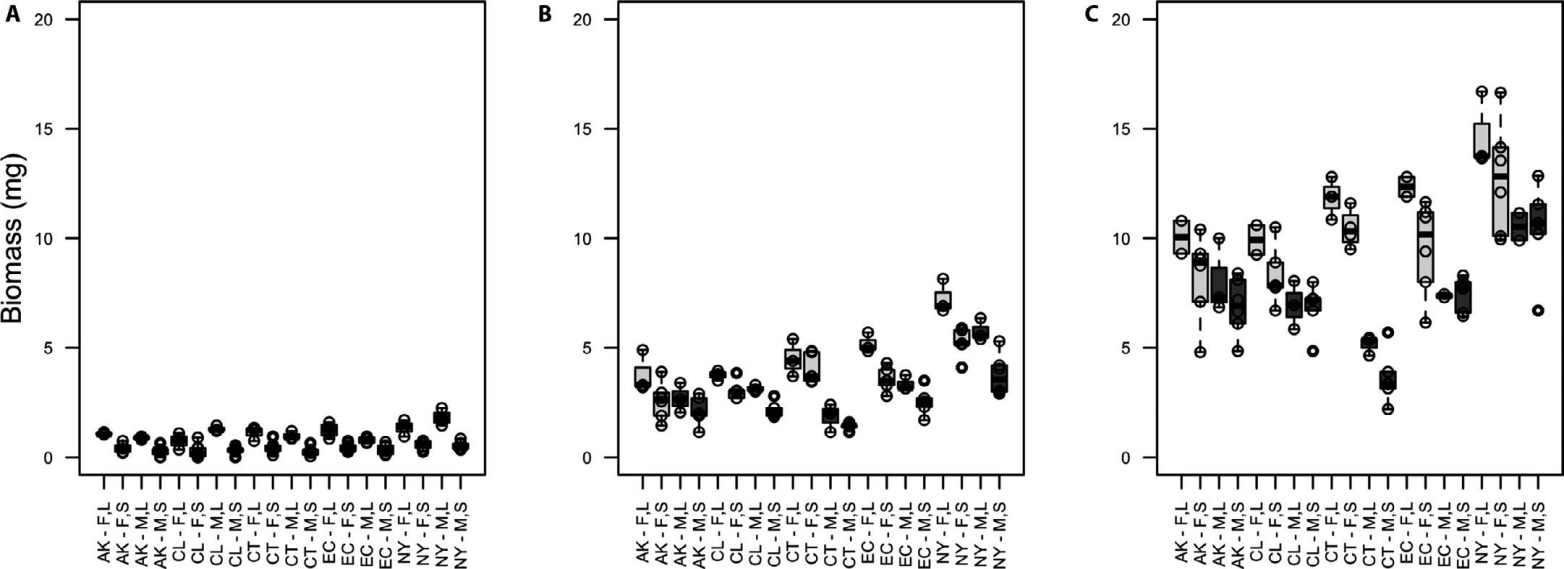
Boxplots of biomass (in milligrams) of *Ceratodon purpureus* isolates after one (A), two (B), and three (C) weeks of growth. Different populations (AK = Alaska, CL = Chile, CT = Connecticut, EC = Ecuador, NY = New York) of female (F) and male (M) isolates at large (L) and small (S) inoculum sizes have a significant difference in biomass at week 1 (A) but show no significant difference in weeks 2 (B) and 3 (C).

**FIGURE 3:**
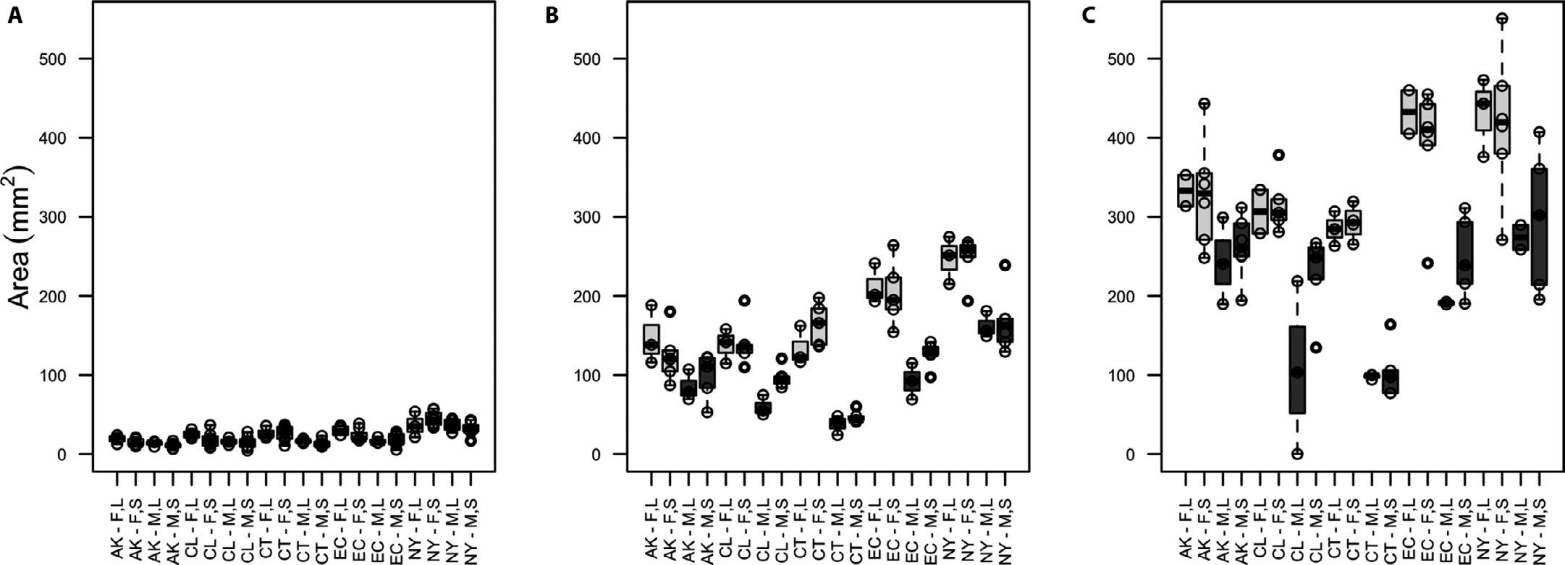
Boxplots of area (in square millimeters) of isolates after one (A), two (B), and three (C) weeks of growth. Different populations (AK = Alaska, CL = Chile, CT = Connecticut, EC = Ecuador, NY = New York) of female (F) and male (M) isolates at large (L) and small (S) inoculum sizes have no significant difference in area at any of the three weeks tested.

To test the effect of inoculum size on area and biomass, we used an ANOVA to compare either area or biomass with inoculum size for each of the three weeks. Inoculum size had a detectable effect on biomass in week 1 (F_1,18_ = 28.51; *P* < 0.0001; Fig. 2A) but no effect in weeks 2 (F_1,18_ = 2.139; *P* = 0.161; Fig. 2B) or 3 (F_1,18_ = 1.127; *P* = 0.302; Fig. 2C). In contrast, inoculum size had no effect on area at any week measured (week 1: F_1,18_ = 0.038; *P* = 0.847; week 2: F_1,18_ = 0.208; *P* = 0.654; week 3: F_1,18_ = 0.088; *P* = 0.77; Fig. 3; data in Appendix S2).

## DISCUSSION

It is common practice to use image analysis of two‐dimensional area as an estimate of growth or fitness in moss protonema. This procedure is non‐invasive, fast, and easy to automate. Nevertheless, the accuracy with which this process estimates biomass, the underlying biologically relevant value for assessing overall growth, has not been critically evaluated. Here we show two‐dimensional area can provide a very good approximation for protonemal biomass, with some caveats. We found a strong and positive correlation between area and biomass after two and three weeks of growth, but not earlier. In addition, the initial size of each replicate (inoculum size) had a negligible effect on that growth. The patterns and associations were also consistent between males and females, despite the obvious sexual dimorphism in protonemal growth rate in this species (McDaniel et al., 2008; Figs. 2, 3). We did find that inoculum size had an effect on biomass at one week of growth, possibly because either we were very close to the range of detection of our scale or because the smaller inocula took more time to initiate growth. However, we found no significant difference in biomass after two weeks. Additionally, we found inoculum size had no detectable effect on protonemal area, indicating that area may be less prone to experimental error than biomass under some circumstances. Therefore, while we still recommend starting with moss inocula of similar size, efforts that aim to minimize size variation at the expense of speed, thus compromising sterility, are not necessary (when variation in size does not exceed 100% from smallest to largest inoculum).

Our study employed a diverse sample from *C*. *purpureus*, suggesting the relationship between two‐dimensional area and biomass is likely to hold true under different conditions and possibly for other species. First, we used paired male and female individuals. In many organisms, later embryonic development is characterized by transcriptional and developmental differences between the sexes, so the fact that the relationship between area and biomass holds for males and females suggests that it may hold across related taxa as well. Second, we included samples in our study that originated from the tropics, the temperate zone, and the high arctic, from populations that are differentiated at other developmental traits. Males and females from tropical to high arctic populations follow the same relationship between area and biomass. In addition, the growth architecture of *Physcomitrella patens* (Hedw.) Bruch & Schimp., the most widely used moss model, is quite similar to *C*. *purpureus* at the protonemal stage. Preliminary observations of *P*. *patens* growth suggest that the relationship between area and biomass may be found in this species too (S.F.M., unpublished data), despite the fact that these species last shared a common ancestor more than 200 million years ago (Laenen et al., 2014).

It is true that some mosses, such as *Aulacomnium palustre* (Hedw.) Schwägr. and *Hylocomium splendens* (Hedw.) Schimp., produce limited protonemal growth and develop gametophores much faster than in either *C*. *purpureus* or *P*. *patens* (W.P.B., S.F.M., and S.B.C., unpublished observations). Such species may require additional care when interpreting area as a proxy for growth. Similarly, some environmental conditions may promote growth forms that are more likely to introduce error in the method that we describe. Elevated humidity, for example, induces vertical growth in some genotypes, and media with decreased calcium concentrations can induce a very compact growth. However, under most circumstances in standard growth conditions when environmental conditions are consistent across samples, two‐dimensional area is likely to provide an accurate estimate of biomass and therefore growth. Thus, taken together, these results will be of use for design and implementation of moss protonemal tissue growth analyses, as well as tissue with a similar growth phenotype.

## AUTHOR CONTRIBUTION

This study was conceived by A.C.P., M.A.L., S.B.C., and S.F.M. The growth experiment was set up by M.A.L. and photograph collection was by A.C.P., M.A.L., and S.B.C. ImageJ analyses were done by M.A.L. Statistical analyses were conducted by S.B.C and W.P.B. Interpretation of results and writing of the manuscript were done by W.P.B., M.A.L., S.B.C., and S.F.M.

## Supporting information


**APPENDIX S1**. Data for area and biomass statistical analyses and scatterplot where replicates within an isolate, inoculum size, and week were averaged.

**APPENDIX S2**. Data for boxplots of area and biomass where replicates within an isolate, inoculum size, and week were not averaged.
